# Sustainability and Scale of the Saving Mothers, Giving Life Approach in Uganda and Zambia

**DOI:** 10.9745/GHSP-D-18-00265

**Published:** 2019-03-11

**Authors:** Jessica Healey, Claudia Morrissey Conlon, Kennedy Malama, Reeti Hobson, Frank Kaharuza, Adeodata Kekitiinwa, Marta Levitt, Davy Wadula Zulu, Lawrence Marum

**Affiliations:** a U.S. Agency for International Development, Lusaka, Zambia. Now based in Monrovia, Liberia.; b Bureau for Global Health, U.S. Agency for International Development, Washington, DC, USA.; c Ministry of Health, Government of the Republic of Zambia, Lusaka, Zambia.; d ICF, Rockville, Maryland, USA.; e HIV Health Office, U.S. Agency for International Development, Kampala, Uganda.; f Baylor College of Medicine Children's Foundation-Uganda, Kampala, Uganda.; g Bureau for Global Health, U.S. Agency for International Development and RTI, Washington, DC, USA. Now with Palladium, Abuja, Nigeria.; h U.S. Centers for Disease Control and Prevention, Lusaka, Zambia. Now retired.

## Abstract

**Background:**

Saving Mothers, Giving Life (SMGL) significantly reduced maternal and perinatal mortality in Uganda and Zambia by using a district health systems strengthening approach to address the key delays women and newborns face in receiving quality, timely, and appropriate medical care. This article documents the transition of SMGL from pilot to scale in Uganda and Zambia and analyzes the sustainability of the approach, examining the likelihood of maintaining positive trends in maternal and newborn health in both countries.

**Methods:**

We analyzed the potential sustainment of SMGL achievements using a tool adapted from the HIV-focused domains and elements of the U.S. President's Emergency Plan for AIDS Relief Sustainability Index and Dashboard for maternal and neonatal health pro-gramming adding a domain on community normative change. Information for each of the 5 resulting domains was drawn from SMGL and non-SMGL reports, individual stakeholder interviews, and group discussions.

**Findings:**

In both Uganda and Zambia, the SMGL proof-of-concept phase catalyzed commitment to saving mothers and newborns and a renewed belief that significant change is possible. Increased leadership and accountability for maternal and newborn health, particularly at the district and facility levels, was bolstered by routine maternal death surveillance reviews that engaged a wide range of local leadership. The SMGL district-strengthening model was found to be cost-effective with cost of death averted estimated at US$177-206 per year of life gained. When further considering the ripple effect that saving a mother has on child survival and the household economy, the value of SMGL increases. Ministries of health and donor agencies have already demonstrated a willingness to pay this amount per year of life for other programs, such as HIV and AIDS.

**Conclusion:**

As SMGL scaled up in both Uganda and Zambia, the intentional integration of SMGL interventions into host country systems, alignment with other large-scale programs, and planned reductions in annual SMGL funding all contributed to increasing host government ownership of the interventions and set the SMGL approach on a path more likely to be sustained following the close of the initiative. Lessons from the learning districts resulted in increased efficiency in allocation of resources for maternal and newborn health, better use of strategic information, improved management capacities, and increased community engagement.

## Abstract (precis)

The Saving Mothers, Giving Life district health systems strengthening approach provides a sustainable model for reducing maternal mortality at scale. Lessons from the learning districts demonstrated increased efficiency in allocation of resources for maternal and newborn health, better use of strategic information, improved management capacities, and increased community engagement.

## INTRODUCTION

Maternal mortality is viewed as a nearly intractable problem in the developing world where the vast majority of maternal deaths occur. When Saving Mothers, Giving Life (SMGL) began in 2012, Uganda's maternal mortality ratio was 310 maternal deaths per every 100,000 live births and in Zambia, 440 maternal deaths per every 100,000 live births.^1^ While the medical interventions to prevent mortality were well known, there was limited evidence that significant reductions in the maternal mortality ratio were possible in the short term. The SMGL initiative hypothesized that a health systems approach would demonstrate significant reductions in maternal and newborn mortality in Uganda and Zambia.^2^ The SMGL approach addressed key principles using interventions based on local context (Box 1). We hypothesized that tailoring interventions to country public health systems and cultural contexts would also enhance sustainability.

BOX 1 **SMGL Primary Principles**

SMGL primary principles include:

1. Surmount the 3 main delays—whether supply- or demand-side—to women receiving lifesaving care
2. Assess and strengthen the existing safe-motherhood safety net in a district, addressing gaps and mobilizing all types of service providers—whether public, private, nongovernmental, or faith-based organization
3. Integrate maternal health care, HIV-related services including prevention of mother-to-child transmission of HIV, and family planning
4. Improve care during labor, delivery, and the first 48 to 72 hours and organize services to ensure access to emergency obstetric care within a 2-hour travel window
5. Capture, analyze, and report all maternal and newborn deaths in a district

SMGL was designed within the context of the Accra Agenda for Action (2008) and publication of the Africa Union's *Campaign on Accelerated Reduction of Maternal, Newborn and Child Mortality in Africa (CARMMA)* (2009) and the World Health Organization's *The Abuja Declaration: 10 Years On*^3^ (2011) and *Beginning with the End in Mind: Planning Pilot Projects and Other Programmatic Research for Successful Scaling Up* (2011).^4^ Initiated within the U.S. Government's Global Health Initiative (GHI), SMGL employed country ownership and strategic coordination/integration as its guiding principles.^5^ It promoted a whole-of-U.S. Government approach to management, incorporating the U.S. Agency for International Development (USAID), U.S. Centers for Disease Control and Prevention, U.S. President's Emergency Plan for AIDS Relief (PEPFAR), Peace Corps, and Department of Defense. From its inception, SMGL was designed to reinforce and strengthen the existing host government health system, build on extant service-delivery platforms—particularly at the district level, and enable countries to achieve their own vision for improved maternal and newborn health.^6^ It was designed to be sustainable and have a clear pathway, through host country systems, to scale. In fact, the majority of the interventions supported by SMGL were not “new” to the host country; rather, they were existing interventions that were refined, strengthened, and, in most cases, taken to greater scale of implementation through partnership. During Phase 1 (2012–2013), SMGL was piloted in 4 districts in Zambia and 4 districts in Uganda—later split into 6 districts in each country—with high maternal mortality. During Phase 2 (2013–2017), the program increased the number of districts to 18 in Zambia and 13 in Uganda.

**FIGURE 1:**
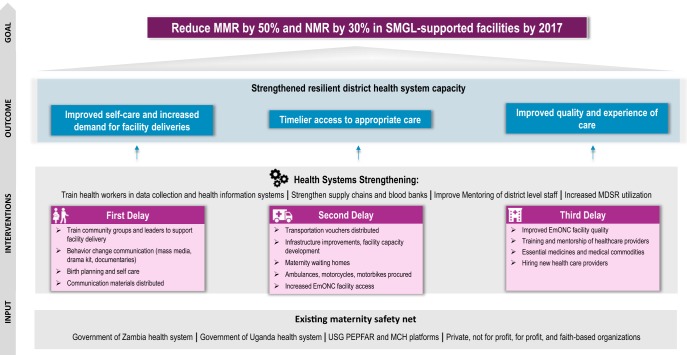
SMGL Theory of Change Model Abbreviations: EmONC, emergency obstetric and newborn care; MCH, maternal and child health; MDSR, maternal death surveillance and response; MMR, maternal mortality rate; NMR, neonatal mortality rate; SMGL, Saving Mothers, Giving Life; USG, U.S. government.

The SMGL theory of change built on a district health systems strengthening approach to surmount critical demand- and supply-side delays that prevent women and newborns from receiving basic and emergency care in a timely manner while also increasing capacity and resilience of the health care system^2^ (Figure 1).

**FIGURE 2:**
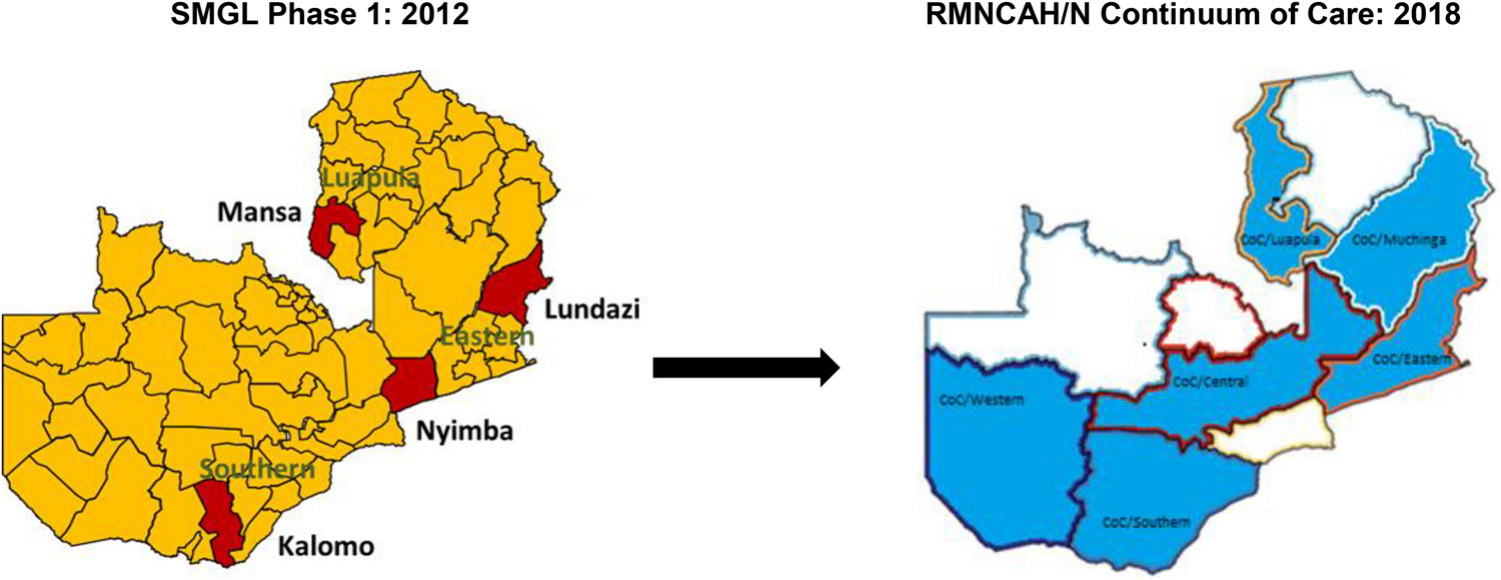
Scale-Up of SMGL Approach in Zambia, 2016–2021 Abbreviations: COC, continuum of care; RMNCAH/N, reproductive, maternal, newborn, child, adolescent health and nutrition; SMGL, Saving Mothers, Giving Life.

The concept of ‘scale’ in this situation—referring to the geographic expansion of the SMGL-supported district-wide approach to maternal, perinatal, and newborn mortality reduction through government and other partner financing—is particularly important given that SMGL began as a proof of concept and, even at the end of the initiative, only covered a small percentage of each country's population. To date, the SMGL approach has been taken to scale in 6 of the 10 provinces in Zambia through the government-led Reproductive, Maternal, Newborn, Child, Adolescent Health and Nutrition (RMNCAH/N) Continuum of Care (CoC) program, covering 53% of Zambia's population (Figure 2). Scaled up through the Ministry of Health (MOH)—with additional direct funding from the Swedish International Development Cooperation Agency (Sida), USAID, and the U.K. Department for International Development (DFID)—the RMNCAH/N CoC program adapts the district- and province-wide health systems strengthening approach with attention to access, demand, quality, and system strengthening and expands focus beyond the 72 hours around delivery to the broader life-cycle for women, adolescents, and children. A majority (80%) of the almost US$125 million total funding (over 5 years-2016 to 2021) were earmarked for direct funding to the districts, with the remaining 20% identified for the province and national levels.

USAID and DFID have procured further technical assistance to continue systems strengthening interventions and focused support to scale up best practices, such as mentorship and data for decision making. SMGL's core interventions (Figure 1) are included in the RMNCAH/N CoC program with a similar approach to first addressing capital investments—such as lifesaving skill development, deployment of skilled birth attendants, infrastructure upgrades, construction of maternity waiting homes, and procurement of equipment and vehicles—followed by a shift in focus in subsequent years to supporting recurring costs, providing strategic mentorship, and conducting outreach. Maternal and newborn health continues to have a significant focus under the CoC program and is the largest technical area of funding, with the majority of districts requesting funds for community-level Safe Motherhood Action Groups (SMAGs), emergency obstetric and newborn care (EmONC) training, maternity waiting homes or staff housing, and mentorship.

In Uganda, scale-up will reach approximately 75% of all districts in 2018 with support from World Bank Global Financing Facility activities; Belgium government-supported maternal, newborn, and child health projects; and USAID maternal and child health programs. The Uganda MOH's *Investment Case for Reproductive, Maternal, Newborn, Child and Adolescent Health Sharpened Plan for Uganda*^7^ drew heavily on the SMGL experience and lessons learned^8^ and will serve as the guiding document for sector investments. “SMGL helped the MOH to take a health systems approach with district leadership,” explains Dr. Jesca Nsungwa Sabiiti of the MOH. At the national level, SMGL provided a testing ground for the Uganda MOH on the impact of providing salary supplements to increase the number of doctors in rural areas. This laid the groundwork for the Wage Bill,^9^ which was aimed at hiring additional doctors at level 4 health center facilities to provide surgical delivery, decongesting district hospitals, and bringing comprehensive EmONC (CEmONC) capacity closer to rural populations. The Wage Bill, put in place in 2016, included allowances to incentivize physicians to serve in rural areas and to improve doctor-to-patient ratios. Related reforms will take effect in 2018 and 2019.^9^

For the Uganda Ministry of Health, SMGL provided a testing ground on the impact of providing salary supplements to increase the number of doctors in rural areas.

**FIGURE 3:**
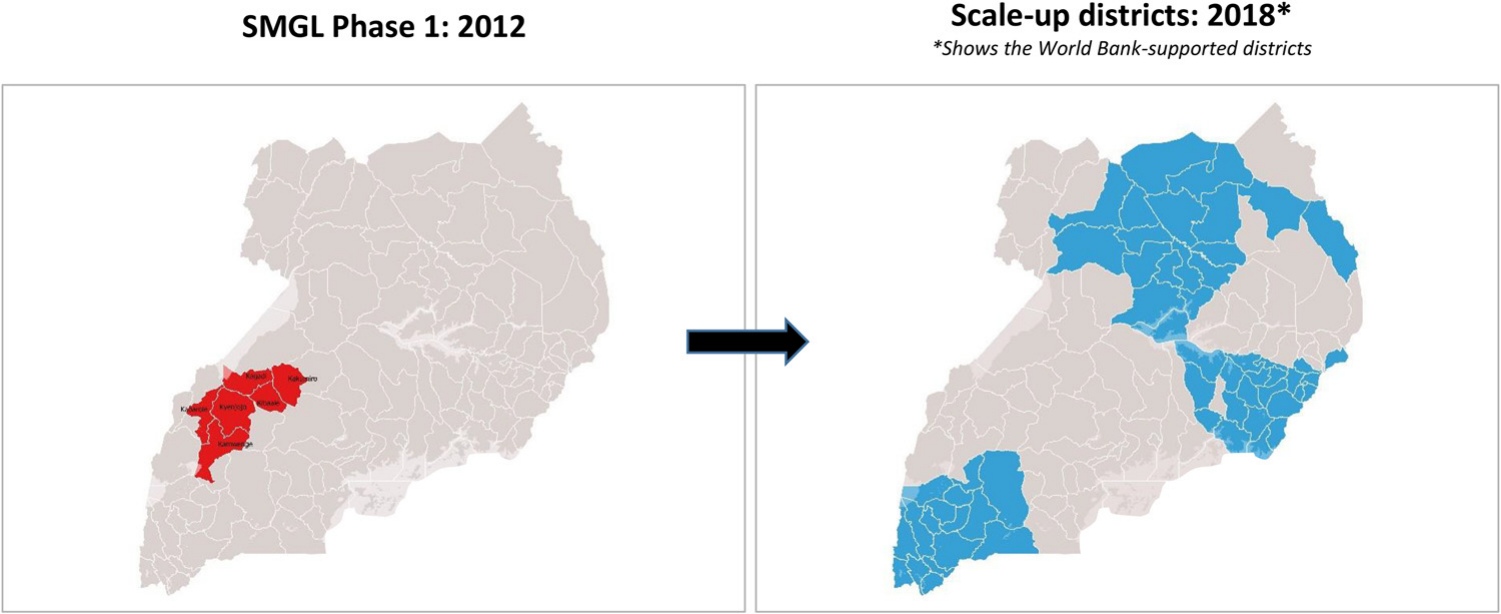
Scale-Up of SMGL Approach in Uganda Through World Bank Support, 2016–2021 Abbreviation: SMGL, Saving Mothers, Giving Life

The Uganda Reproductive, Maternal, and Child Health Services Improvement Project,^10^ developed in 2016 by the World Bank and launched in 2017, will take the SMGL health systems approach to scale in 80 of Uganda's 121 districts (Figure 3). Another World Bank-supported program, the Uganda Reproductive Health Voucher Project, includes a modified version of the piloted SMGL program that provides vouchers for poor women to access safe delivery.

**FIGURE 4:**
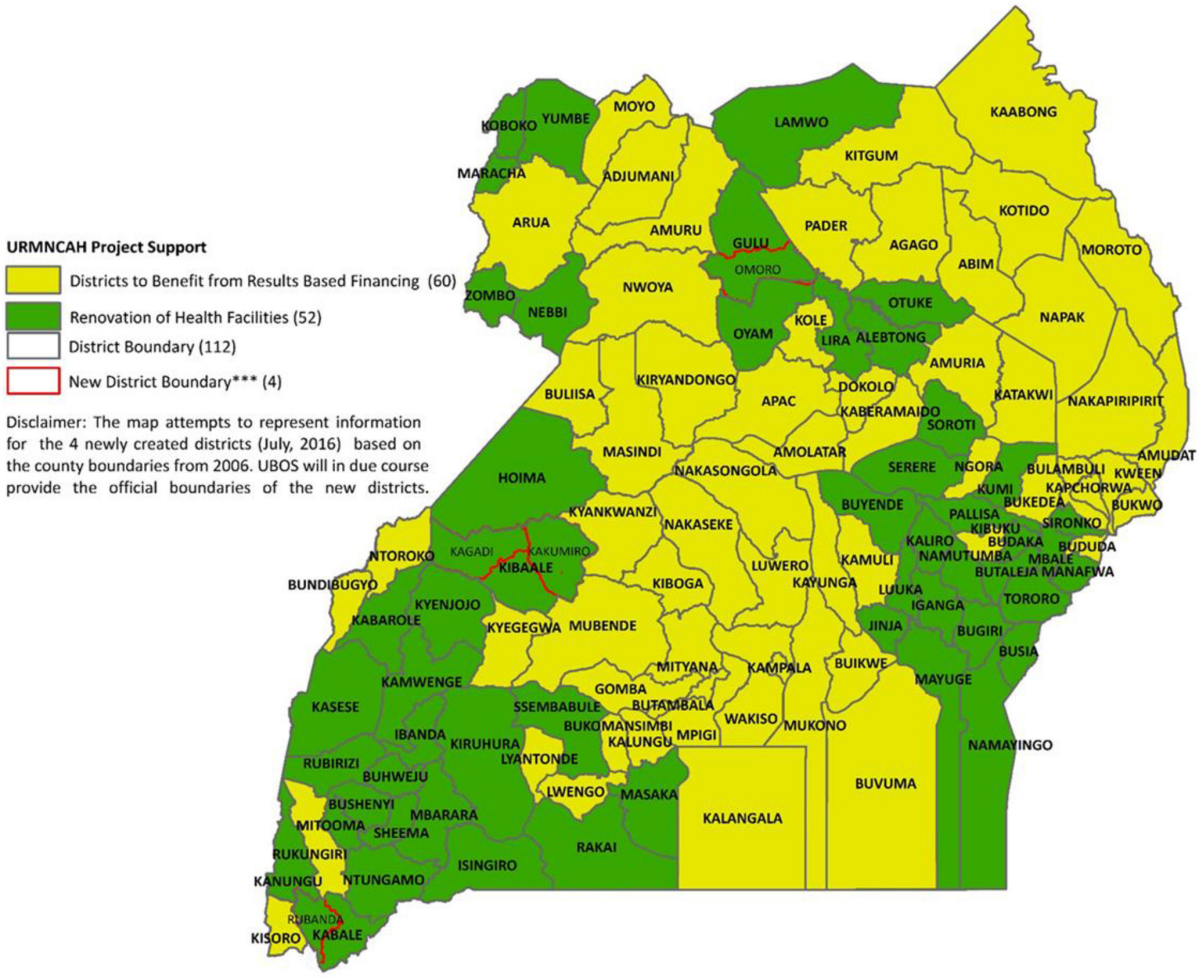
Scale-Up of SMGL Approach in Uganda Through Regional Health Integration to Enhance Services, 2012–2022 Abbreviations: UBOS, Uganda Bureau of Statistics; URMNCAH, Uganda reproductive, maternal, newborn, child, and adolescent health.

The USAID-funded Regional Health Integration to Enhance Services project,^11^ covering 61 districts, and Belgium Government investments^12^ in maternal and child health, similarly built on core components of the SMGL approach through results-based financing (Figure 4).

In 2014, Nigeria was added as the third SMGL country with implementation continuing through September 2019. Although this article does not assess the prospective sustainability in Nigeria directly, it documents the global scale of the SMGL approach in Cross River State, which is composed of 18 local government authorities with a total population of 3.7 million. The SMGL ‘whole market’ or health systems strengthening approach is employed with involvement of both public and private providers and supported by funding provided from Cross River State, USAID, and Merck for Mothers. Geographic information system travel-time mapping was used to select EmONC facilities that needed to be upgraded in order to increase women's access to lifesaving care in their catchment areas. Since SMGL entered Phase 2 in 2014, other countries, including Afghanistan, have redesigned their maternal and newborn programs based on the SMGL approach.^13^

In the context of the significant scale-up of the SMGL approach in Uganda and Zambia, we analyzed the likelihood that the approach will be sustained and, in turn, that the encouraging results will continue on a larger scale.

### Method: Sustainability Framework

The SMGL design and approach was influenced by existing global thinking, with strong emphasis on reducing maternal and newborn mortality in Africa, increasing country ownership, and strengthening local capacity.^14^^–^^17^ In 2009, the U.S. Government launched GHI, which characterized country ownership as the ability of the government, communities, civil society, and private sector to lead, prioritize, implement, and be accountable for a country‘s health response as outlined by a 4-dimensional framework: (1) political leadership and stewardship, (2) institutional and community ownership, (3) capabilities, and (4) mutual accountability, including finance. Since that time, ideas related to country ownership and capacity strengthening have evolved and focused on the ultimate goal of sustainability beyond foreign assistance. Our focus on sustainability looks at whether the results of SMGL, particularly maternal and perinatal mortality reduction, will continue at similar rates after the initiative and whether the approach will continue to be used in host country systems.

SMGL was designed based on the GHI principles and built on PEPFAR's foundational work and partnerships in-country during that period.^18^^,^^19^ Our primary question was: If we can be so successful in reducing deaths to people living with HIV and AIDS, why not for mothers giving birth?

SMGL was designed based on the GHI principles and built on PEPFAR's foundational work and USG partnerships in-country.

In 2014, PEPFAR developed the Sustainability Index and Dashboard (SID) that covers 4 domains of sustainability: (1) governance, leadership, and accountability; (2) national health systems and service delivery; (3) strategic investments, efficiency, and sustainable financing; and (4) strategic information.^6^ Although designed for HIV and AIDS, SID provides a familiar framework for assessing sustainability and supplements global and host country literature in this area.^20^^,^^21^ We modified the SID domains with questions specific to maternal and newborn health and added a domain of “community normative change” to assess the prospective sustainability of SMGL interventions in reducing maternal and newborn mortality along the 3 delays. The added domain measured social and behavior change, demand for quality services, and the role of local leaders and champions in influencing change (Table 1).

**TABLE 1.** SMGL Sustainability Index Domains and Key Questions

Domains	Prompts/Questions for Ministry of Health Staff, Leadership, and Decision Makers at National, Provincial, and District Levels
Community normative change Behaviors Demand for quality services Social norms	How has the % of deliveries in health facilities changed? Is there a change in proper use of and demand for waiting shelters? What % of women/families had a birth preparedness plan, saved money, and pre-arranged for transportation? How has the use of vouchers in Uganda changed and been institutionalized? What is the evidence of local customs/norms changing? How has male engagement in birth planning and maternal health changed? What is the sustained level of engagement of community health cadres for normative change (SMAGs in Zambia, VHTs in Uganda)? Is there evidence of prolonged leadership of “change champions” in the community?
Governance, leadership and accountability Willingness to champion change Planning/coordination Policies and governance Civil society engagement Private-sector engagement Public access to information	Are there national or local champions that emerged from SMGL who successfully advocate for improved maternity services? How has SMGL influenced changes in government policies and guidelines that are critical to long-term improvements in maternal and newborn survival? At the national level, which guidelines, policies, or tools were updated? Has the implementation of policies been institutionalized at the lower level to sustain the benefits to maternal and newborn health? Has the role of the community workers/VHTs in ensuring women are linked to appropriate care been institutionalized? Will the role of the private sector in providing maternal and newborn health services continue after SMGL? Has the government established public–private partnerships? What evidence exists of change in public access to information on maternal and newborn health at the district level or below? Has the role of the community workers/VHTs in ensuring women are linked to appropriate care continued after SML?
Health system and service delivery Service delivery Human resources for health Commodity security Quality management	Have signal functions—such as newborn resuscitation, administration of anticonvulsants and oxytocics, cesarean section, and manual removal of placenta for EmONC and CEmONC—been institutionalized? Has the government scaled up the district systems strengthening approach/key components of SMGL? Which components has the government picked up? Has there been a transition of SMGL-supported human resources to government positions or has the government at the district level started to fund the SMGL-contracted positions? To what extent? Has the government picked up the funding of lifesaving drugs such as oxytocin and commodities such as balloon tamponades or anti-shock garments to prevent and or treat postpartum hemorrhage and eclampsia? Has the government institutionalized some type of district/health facility assessments/quality assurance approach to use as the basis of planning and budgeting? Is the blood supply for transfusion adequate? Is fresh frozen plasma available?
Strategic investments, efficiency, and sustainable financing Domestic resource mobilization (capital investments and recurring costs) Technical and allocative efficiencies	Has there been an increase in domestic financial resources for maternal and newborn health in SMGL-supported districts to continue the quality of services? Has the government budgeted and allocated funding for the scale-up of the SGML approach in other districts? Have they included funding considerations for both capital investments and recurring costs? What key components were taken to scale by the government? What components of SMGL were eliminated or reduced as they were not affordable or cost-effective? Was there any study on efficiency or cost-effectiveness? Did SMGL influence planning of Ministry of Health resources or improve technical/allocative efficiencies?
Strategic information Epidemiological and health data Financial/expenditure data Performance data	Were maternal death audits institutionalized? Were data reviews institutionalized? After SMGL, how are districts/facilities continuing to use data to improve maternal and newborn outcomes?

Abbreviations: CEmONC, comprehensive emergency obstetric and newborn care; EmONC, emergency obstetric and newborn care; SMAG, Safe Motherhood Action Group; SMGL, Saving Mothers, Giving Life; VHT, village health team.

To collect information on sustainability, 86 key informants from the SMGL-supported districts in both Uganda and Zambia, the countries' respective ministries of health, the U.S. Government, and implementing partners were interviewed individually, in person or by phone, or in group discussions (Table 2). Key informants came from a range of SMGL districts, and additional in-depth interviews were held with select informants from the original Phase 1 SMGL districts. In addition to reviews of key stakeholder interviews, data from the health facility assessments (HFAs) on capacity and readiness of the system to provide EmONC signal functions were extracted to provide a clear understanding of the existing maternal health safety-net in the original SMGL-supported districts in each country. HFAs were carried out at 3 time-points: (1) at baseline in 2012, to inform SMGL planning and design and needed investments; (2) at the end of the pilot year in 2013, to gauge progress and inform funding and operational decisions during years 2 to 4; and (3) at initiative endline in late 2016, to assess outcomes. After assessing the 5 domains of sustainability, major findings were organized into the main SMGL focal areas of demand for care, access to care, quality of care, and overall health systems strengthening.

**TABLE 2.** Key Informants for SMGL Sustainability Domains

Stakeholders	KIIs on Sustainability of SMGL (No.)	Participants in Group Interviews on Sustainability (No.)
**U.S. Government, field**		
Uganda	4	0
Zambia	6	0
**Host government, national**		
Uganda	4	3
Zambia	2	0
**Host government, subnational**		
Uganda	7	0
Zambia	4	38
**In-country partner**		
Uganda	7	8
Zambia	5	0
**Total**	**39**	**49**

Abbreviations: KIIs, key informant interviews; SMGL, Saving Mothers, Giving Life.

## RESULTS

The most salient findings from the data review and stakeholder interviews across adapted SID domains are presented here.

### Community Normative Change

In several languages in Uganda and Zambia, the greeting of a mother who has given birth includes an element of surprise and relief that she has survived the perils of childbirth: in Bemba, *Mwapusukeni* (“You have survived”); and in Luganda, *Kulika omwana* (“Thank God you have survived with this baby”). Overcoming fatalism and encouraging confidence in a health care system that can respond to the complications related to birth is an important step in increasing demand for facility-based deliveries. Also essential are skilled and competent birth attendants and communities that are engaged to champion this change. The most significant evidence of sustainability in this domain is the formalization and institutionalization of robust community volunteer groups to champion maternal and newborn health; continuation of activities and leadership for maternal health from change champions; and proliferation of maternity waiting homes by diverse funding actors in collaboration with local communities in Zambia. Birth plans may be sustainable, but it is too early to tell if host country governments will continue to print and distribute them, despite interest in and current commitments to doing so. In Uganda, vouchers were not directly sustainable but the findings from the voucher pilot will inform larger social protection schemes in the country.

Overcoming fatalism and encouraging confidence in a health care system is an important step in increasing sustained demand for facility-based deliveries.

SMGL worked with ministries of health and other partners to leverage and strengthen predominantly effective existing interventions and promoted change champions, community volunteer groups for education and referrals (SMAGs in Zambia and village health teams [VHTs] in Uganda), and improved access to delivery services through a system of vouchers in Uganda and maternity waiting homes in Zambia.

**Figure:**
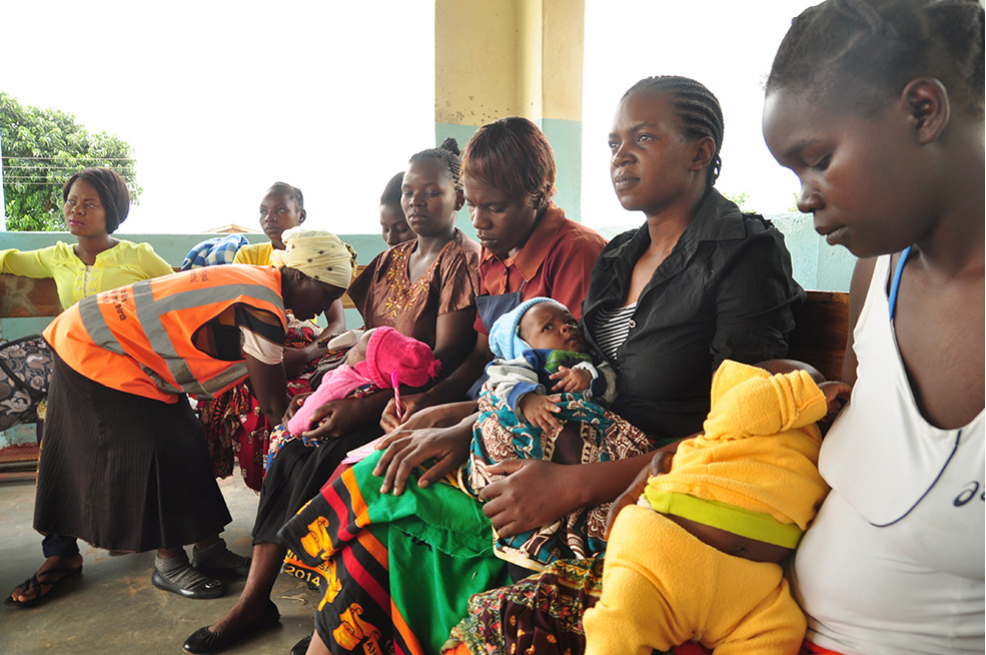
Mothers wait with Safe Motherhood Action Group member at Lundazi Urban Health Centre, Zambia. © Amy Fowler/USAID

At the national level, both Uganda and Zambia expanded the formal role of community change agents for maternal survival by formalizing guidelines and training packages for SMAGs and VHTs, which were implemented in both SMGL and non-SMGL districts. SMGL also ensured that tested materials for birth planning and outreach—radio spots, local language documentaries, and counselling aids for community leaders—were made increasingly available.

The most significant findings, however, were at the district level and below, stimulated by increased information on safe motherhood at the local level and the transfer of ownership of maternal health to traditional leaders, communities, and women and their families (Table 3).

**TABLE 3.** Indicators and Data on Community Norms

	Uganda	Zambia
% change in institutional delivery rate (2012–2016)	47% increase (from 45.5% to 67%)	44% increase (from 63% to 90%)
# trained SMAGs/VHTs reporting to health center (cumulative individuals trained from baseline)	11,189	13,658
# of change champions	N/A	350^23^
% of all institutional deliveries in SMGL-supported original districts supported by transport vouchers (2012–2016)	24%	N/A
% change in number of facilities with a maternal waiting shelter from 2012 to 2016	N/A	69% increase

Abbreviations: HFA, health facility assessment; N/A, not available; SMGL, Saving Mothers, Giving Life; VHT, village health team.

^a^Data from HFAs unless otherwise noted.

In both countries, change champions, particularly traditional leaders and chiefs in Zambia and district local councils in Uganda, were active and, according to multiple informants, directly increased male involvement in birth and delivery preparedness. This support continued regardless of availability of financial support from SMGL and was cited in focus group discussions as an important factor for sustainability. The district health office in Kalomo, Zambia, confirmed, “Now every time Chief Chikankata has a meeting with Headmen, he asks us to come. Previously these were only ‘women's issues’ but now everyone is involved. The male engagement has meant more women are delivering at facilities and has even reduced child marriage.” Local leadership said this also influenced demand for family planning from women and support from their husbands. In some cases, local ownership resulted in unintended consequences, such as chiefs developing by-laws requiring antenatal care and fining male members of the household who allowed women to deliver at home without a justification (Kalomo and Nyimba). While this was incorrectly attributed directly to the SMGL intervention, it was a sincere reflection of local ownership and embedding of SMGL activities into community structures, which undoubtedly increased antenatal care and facility deliveries.^2^

Printed birth plans under SMGL aimed to increase birth preparedness and awareness of danger signs, which contributed to the significant demand for maternity waiting homes by both women and district leadership.^22^^–^^25^ Birth plans provide a facilitated opportunity for health care providers to discuss pregnancy, delivery, and postnatal care and to plan for social support, logistics, nutrition, and care with pregnant women and their partners. The results of a birth plan audit conducted by Communications Support for Health estimated that 139,200 people in Zambia were exposed to the birth plan. Results from a random sample stated that over 70% of respondents recalled having seen the birth plan: 51% of women said they used the birth plans to learn about danger signs, with 48% saying they used it to prepare logistics and 43% saying they used it to remind them to save money. Birth plans included smiley and frowny faces that allowed women and families to assess their experience of care as a feedback tool for facility staff.

While not unique to SMGL, the scale of distribution of birth plans, mentoring of health workers to use the birth plans for counseling, and systematic inclusion in the package of support for safe motherhood made the tool a routine part of facility outreach and counseling and, as demand for services increased, was appreciated and requested by expecting mothers and facility staff. District MOH leadership in Zambia, recognizing the importance of the plans, committed to continued printing and distribution beyond SMGL.^24^

In Uganda, the transport voucher system pilot implemented during SMGL successfully enabled women to deliver their babies at facilities and facilitated adherence to the national plan of 4 antenatal visits, while addressing the first and second delays. Because it is not inherently sustainable without being embedded in another program, the voucher system will be scaled up under the World Bank's Uganda Reproductive Health Voucher Project and is being considered as a component of the national health insurance scheme. In Zambia, maternity waiting homes were the MOH's central tool for increasing access to health facility delivery and were cited as in high demand by all districts. Waiting shelters were associated with 49% of facilities by 2016, which was an increase of 69% from the baseline. District health officials cited these shelters as contributing to sustained access to care but highlighted concern that demand would soon exceed supply if the government was not able to continue new shelter construction. Communities and districts are now requesting new maternity waiting homes through other donor support and continuing to contribute materials and labor through communities.^25^

In Uganda, the transport voucher system pilot enabled women to deliver their babies at facilities and facilitated adherence to the national plan of 4 antenatal visits.

For sustainable changes in community norms, several informants highlighted the need to stay vigilant in reaching local leaders and influencers with locally relevant information. In Uganda, this included active engagement of traditional birth attendants as village health workers (VHWs). In Zambia, sustainable changes included maintaining incentives and support structures for SMAGs and traditional leaders, particularly as the approach extends to new districts. Routine costs of VHWs and SMAGs have been largely absorbed by the host governments and other donor partners but will need continued oversight to ensure sustainability, especially for larger purchases such as bicycles. In Zambia, districts involved in both SMGL and the CoC program began budgeting for SMAG training, monthly meetings, and SMAG incentives from their own government grants and from donor funding. Engaging traditional leaders by, for example, paying honoraria as was done under SMGL in Zambia, was considered costly and controversial but was still determined to be a critical investment and a powerful and sustainable tool for shifting norms. Several district and provincial health officials in Zambia stressed the need for long-term sustainability plans to tackle cross-sectoral challenges that were beyond the remit of SMGL—such as roads, literacy, and poverty—in order to continue making significant progress in maternal and newborn health. Host countries and funding partners need to better coordinate investments to address the thorny underlying roots of poor maternal and newborn health to catalyze a more rapid decline in maternal and perinatal morbidity and mortality.

### Governance, Leadership, and Accountability

Multiple respondents echoed the sentiments on increased ownership and leadership of maternal health issues that the external evaluation of SMGL captured in 2013: “[Before SMGL] there were many mothers dying in silence. At least now when mothers die, people notice, and they try to learn from it. It's a big issue. Now when a mother dies, we know before lunch.”^26^ Ministry of Health district leadership noted that SMGL created champions and leaders—well beyond just medical professionals—for maternal health across the district. Increased visibility and leadership and greater accountability were the most notable findings within this domain. This was exemplified in the maternal death review process in both Uganda and Zambia, which started by bringing in key stakeholders under SMGL, with health sector officials and communities identifying roles each can play in reducing maternal mortality and, in Zambia, with the district commissioners chairing this committee. This is now a routine process for maternal death reviews, an important factor for districts sustaining ownership and action on findings.

At the national level, SMGL incorporated and codified evidence-based interventions through new and updated policies, guidelines, and training materials (Box 2). These core documents are used routinely for planning and training and will continue as guiding documents for the health sectors in both countries. In Uganda, an MOH official noted that “the Ministry of Health sees SMGL as a learning opportunity for the rest of the system.”^26^ This was realized over the course of SMGL in both countries through testing improvements to change policies, tools, and systems.

BOX 2 **Guidelines, Policies, and Training Materials Developed or Updated Under SMGL**

Zambia

- Clinical mentorship guidelines
- Quality improvement guidelines
- Maternal and perinatal death surveillance and response
- EmONC training (revised to include UBT placement)
- SMAG training
- Every Newborn Action Plan
- Neonatal management guidelines

Uganda

- Clinical mentorship guidelines (AOGU)
- Maternal and perinatal death surveillance and response
- Perinatal death surveillance and response (BABIES matrix)
- Essential Training in Operative Obstetrics (from ACOG/AOGU partnership)

Abbreviations: ACOG, American College of Obstetricians and Gynecologists; AOGU, Association of Obstetricians and Gynecologists Uganda; BABIES, birth weight and age-at-death boxes for an intervention and evaluation system; EmONC, emergency obstetric and newborn care; SMAG, Safe Motherhood Action Group; UBT, uterine balloon tamponade.

The ability of SMGL to mobilize and sustain public–private partnerships did not prove to be as sustainable and scalable as anticipated. It was difficult to attract new global partners after the decision was made, at the end of Phase 1, to select only 1 additional sub-Saharan African country, Nigeria. In Zambia, while 2 local partners, LaFarge Cement and Stanbic Bank, joined SMGL and made important contributions, they have not yet committed to longer-term agreements beyond their initial corporate social responsibility investments. However, there was increased awareness of maternal health issues among these large companies that demonstrated the pull of the success of SMGL to bring new players into the big push for reducing maternal mortality.

The ability of SMGL to mobilize and sustain public–private partnerships did not prove to be as sustainable and scalable as anticipated.

### Health System and Service Delivery

At the national level, the revised policies and guidelines in both countries benefited districts beyond SMGL and will be cornerstones as the approach continues to national scale. Significant outcomes included introducing uterine balloon tamponade training and other skills sessions in the EmONC curriculum in Zambia to better control postpartum hemorrhage, which is the leading cause of maternal mortality. A significant shift from offsite training to onsite mentorship programs was made in both countries, which became the host governments' core approach. In Zambia, the high-frequency/low-dose method of mentorship was supported because it was positive and encouraged health workers while previous mentorship approaches had been viewed as negative and punitive. Tackle boxes for postpartum hemorrhage and eclampsia were innovations provided to health workers, based on the mentorship experience. In Uganda, most notably, the health professional associations took the lead in providing mentorship to health workers. Routine mentoring visits have been reported as an effective tool to increase knowledge and improve practical skills among health professionals.^27^ Mentorship is less costly than retraining staff and helps develop a greater culture of shared responsibility between levels of the health system. As a formal tool used by the ministries of health, mentorship also led to local innovation. A former provincial health director in Zambia, Dr. Mathew Ngambi, described how doctors and midwives in Luapula Province formed WhatsApp groups for continued mentorship and provision of advice with a focus on maternal health. While conducting drills on treating eclampsia and postpartum hemorrhage, a mentor in Southern Province noted the time it took as the midwife rushed to various places in the health center to gather supplies, medications, and fluids. In response, the mentor designed tackle boxes containing all the essential supplies for these 2 conditions to be placed in arm's reach of the delivery table.

To further bolster training and mentoring and ensure knowledge retention, availability of updated protocols and guidelines is also crucial; HFAs reported availability increased at almost all facilities in Uganda during implementation of the SMGL initiative. For topics such as eclampsia and magnesium sulfate, postabortion care, and postpartum hemorrhage, the increase in availability of protocols and guidelines from baseline to the end of Phase 2 was significant, increasing from 9% to 74%, 8% to 50%, and 15% to 86%, respectively. We posit this will bolster long-term improvements in service delivery.

In both Uganda and Zambia, HFA results demonstrated that commodity security improved despite ongoing challenges. In Uganda, essential medicines, such as oxytocin and magnesium sulfate, became increasingly available at public health facilities during the 12 months preceding the HFA (2013 and 2017), although some essential antibiotics, such as gentamicin, and other routine antenatal care medications were less available during the same period. Zambia also saw improvements across several key drug availability indicators, although the overall picture was mixed. Hospitals had higher drug and HIV test kit stock rates, but one-quarter of the hospitals faced stock-outs for oxytocin. For medicines such as gentamicin and magnesium sulfate, availability improved at the end of Phase 1 but was reduced in Phase 2. Decreasing trends between end of Phase 1 and endline for several service delivery indicators might indicate that the focused and well-funded programming during Phase 1 was not yet institutionalized across districts and facility types, as funding decreased during Phase 2. These mixed results show the strategic value of leveraging existing supply chain systems and the impact of increased accountability for maternal mortality on the larger health system—since SMGL did not directly support the supply chain—in both countries. It also illuminates an area where additional focus may be required in order to truly sustain achievements (Table 4).

**TABLE 4.** HFA Data on Health Systems and Service Delivery in Original 8 SMGL Districts, 2012–2016

	Uganda		Zambia	
	Baseline	Endline	Baseline	Endline
Infrastructure – facilities with electricity	56%	96%	57%	93%
Infrastructure – facilities with running water	75%	100%	90%	97%
No stock-out of medicines – oxytocin	56%	82%	75%	75%
No stock-out of medicines – magnesium sulfate	48%	64%	20%	43%
Population-based cesarean delivery rate	5.3%	9.0%	2.7%	4.8%
24 hours a day/7 days a week services at facilities	78%	89% (NS)	65%	96%
Facilities with available transportation (vehicle or motorcycle)	61%	59% (NS)	55%	73%
Facilities with communications equipment	93%	99%	45%	100%

Abbreviations: HFA, health facility assessment; NS, not significant; SMGL, Saving Mothers, Giving Life.

SMGL's direct financial support for capital investments in health facility infrastructure—such as increasing access to running water, electricity, and communications—and skill-building was an important factor for improving confidence in the health system and providing critical support to district and provincial health directors. Such investments are likely to have a long-term impact on service quality and availability, as this type of system strengthening can endure well beyond SMGL and translate into progress in other health areas. In focus group discussions, district and provincial leadership said that their staff and budgets were sufficient to maintain the current infrastructure investments to date; however, this deserves continued attention moving forward. Although further capital investments are planned under donor-supported scale-up activities in both Uganda and Zambia, it is unlikely that the host governments, without support, would continue those investments in the near-term. The number of facilities providing the 7 basic EmONC (BEmONC) signal functions increased from 3 at baseline to 8 at endline in the 4 district-regions in Zambia and from 3 to 9 in the 4 districts in Uganda. Using the World Health Organization minimum recommended level of 5 EmONC facilities per 500,000 population, 3 of the 4 original SMGL districts in both Uganda and Zambia achieved the recommended minimum. Similarly, facilities providing 9 CEmONC signal functions increased from 4 to 5 facilities in Zambia and 7 to 17 facilities by endline in Uganda across SMGL districts.

The number of facilities providing the 7 BEmONC signal functions increased from 3 at baseline to 8 at endline in Zambia and from 3 to 9 in Uganda.

Capacity to provide a blood transfusion is essential for surgical delivery and resuscitation for severe obstetric hemorrhage and is a CEmONC signal function. To expand the number of facilities with CEmONC capacity in Uganda, SMGL provided blood bank refrigerators to level IV health centers and transported blood from the regional blood banks to these facilities. SMGL provided training for lab technologists on blood grouping and cross matching and for doctors and nurses in prescribing and delivering blood transfusions. In Zambia, where over one-third of blood transfused is for pregnant women, an increased supply of blood was provided to SMGL-supported districts and a pilot initiative began to provide fresh frozen plasma—which has a 1-year, compared to 1-month, shelf life and does not require cross matching—to selected health centers. The 2016 HFA in Zambia, showed that blood transfusion capabilities were maintained in all districts except for Kalomo, which lost this capacity when Zimba district was split off and the remaining district lacked a functioning operating theater. Since 2016, there have been important improvements in the district including increased training, new full-time district leadership, and completion of the only surgical theater in the district. The Zambian government has increased funding to the National Blood Transfusion Service, as external donor funds declined, and plans to develop blood banking hubs nationally in 2018–2019, which may address some of the constraints.^28^ Inadequate blood supply, however, continues to be a challenge that needs additional funding in both Uganda and Zambia to reduce maternal deaths.

Human resources for health is an area that will continue to require significant support post-SMGL despite substantial strides made, particularly in Uganda. In Uganda, facilities were supported under SMGL to reduce vacancy rates and recruit health staff—at the government rate—who would be absorbed with the increase in salaries as stipulated in the Wage Bill^9^ when it comes into effect in 2019. Almost three-quarters (74%) of health workers hired directly by SMGL in Uganda were eventually absorbed into the health system and all those staff continue to be paid by the government at salary levels stipulated in the national policy. This was an important boost for the sustainability of activities and availability of service providers in the associated facilities. In Zambia, “retired but not tired” midwives were hired directly by SMGL for health centers that lacked a nurse or midwife; however, they could only be given 1-year renewable contracts due to retirement laws and regulations. Enrolled nurses were given additional midwifery training, in part due to lessons learned from SMGL. As Zambia works to expand the pipeline of skilled health workers, SMGL highlighted the opportunity for the government to consider further involvement in and formalization of hiring options for the skilled but retired or out-of-work midwife cadre to support health services.

**Figure:**
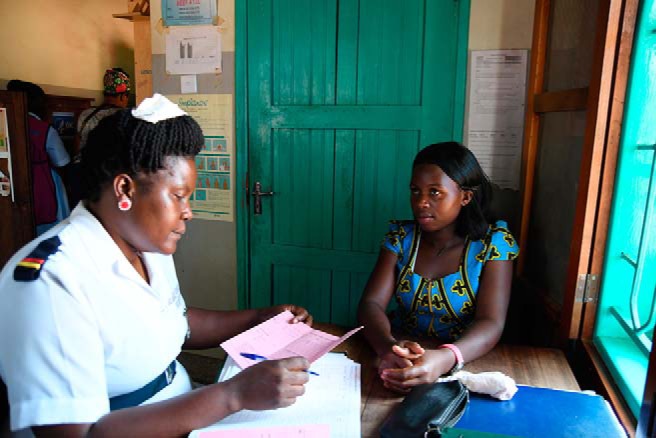
A service provider examines a client at the SMGL-supported Kakumiro Health Center in Uganda. © Amy Fowler/USAID

Overall analysis of the sustainability domain questions in the HFA reports demonstrated significant improvements and prospects for sustainability across indicators for human resources, health facility infrastructure, and access to EmONC services in both Uganda and Zambia.^28^ There was some reduction in performance of some indicators following Phase 1, when there was a funding gap of 1 year due to a significant break in implementing partners in both Uganda and Zambia due to procurement timelines as well as an extended delay in funding reaching both countries. During Phase 2 there was a planned reduction in funding during the second year by 25% from the first year, a 50% reduction the following year, and a 75% reduction in the final year to naturally push for greater government ownership. These circumstances provided an opportunity to demonstrate the sustainability of interventions and the ability of local and national governments to assimilate activities from the “big push” into national maternal and newborn health programs. Through this lens, Uganda had greater success in sustainable improvements for human resources for health. However, in both countries, early investments in infrastructure are likely to continue to pay dividends in access to quality care and services.

### Strategic Investments, Efficiency, and Sustainable Financing

While SMGL was not designed to specifically increase national maternal health budgets, we hoped that the programmatic results would drive governments to increase domestic resource allocation for continued quality maternal and newborn services.^8^ In the end, SMGL did not contribute to significant changes in either country's domestic resource mobilization or increased government funding. SMGL maximized allocative efficiency by identifying high-impact activities that, when clustered, addressed the 3 delays and provided health system benefits to population groups beyond mothers and newborns, such as emergency transportation, improved infection prevention, water supply and electricity at health facilities, and availability of surgical services and operating theaters—all critical components of a functioning health system. SMGL districts in Zambia cited a significant change in the district's annual plans for maternal health. Several districts cited the value of a ‘blueprint’ for health systems areas of improvement and, in particular, cited an important increase in planning and prioritization around the first and second delays (demand and access to services). In Zambia, for example, multiple district partners highlighted the increased investment in community structures and health promotion to increase demand. This approach was recently codified with requirements from the MOH that 10% of all budgets must directly benefit the community. In Zambia, SMGL funds were channeled directly to 2 of the 4 focus provinces that oversaw implementation in the SMGL districts—approximately US$300,000 per province per year—from the U.S. Government and Sida (for 1 year) to support the SMGL approach. This direct support further engaged the districts and resulted in a natural transfer of central activities from SMGL support to direct funding. As already noted, SMGL directly influenced greater investments under the CoC program in Zambia and the World Bank Reproductive, Maternal, and Child Health Services Improvement project in Uganda, leveraging 5-year investments of US$125 million in Zambia and US$140 million in Uganda. In Zambia, this translated into prioritizing interventions under SMGL for direct funding from donors and direct host-country resources and, in Uganda, the Sharpened Plan Investment Case^7^ for RMNCAH built heavily on the SMGL approach.

SMGL maximized allocative efficiency by identifying high-impact activities that, when clustered, addressed the 3 delays and provided health system benefits to population groups beyond mothers and newborns.

A separate study in this supplement by Ben Johns et al.^29^ was conducted to determine costs—incremental costs and incremental cost per death averted—of the SMGL district strengthening approach. They found that the incremental cost for maternal and newborn care per SMGL-supported district in 2016 was US$845,000 in Uganda and US$760,000 in Zambia. This translates into about US$38 per facility birth in Uganda and US$95 per facility birth in Zambia in 2016. In Zambia, the cost per death averted was US$12,514, or $206 per life-year saved. In Uganda, the cost per death averted was US$10,311, or $177 per life-year saved. The researchers concluded that the approach was cost-effective, with the cost of life-year gained as a percentage of gross domestic product (GDP) being 26% and 16% in Uganda and Zambia, respectively. The authors concluded that the SMGL program “could be paid for by increasing health spending from 7.3% to 7.5% of GDP in Uganda or 5.4% to 5.8% of GDP in Zambia.” This is far less than domestic investments for other health areas, such as HIV and AIDS.^30^ The analysis did not take into consideration the costs associated with the considerable ripple effects a maternal death has on children, families, and communities.^31^^,^^32^

The district strengthening approach, as demonstrated in SMGL, represents a substantial cost-effective health investment, one that low- and middle-income countries can afford.^29^

### Strategic Information

At the national, provincial, and district levels, improvements in strategic information were viewed not only as sustainable systems-level improvements in the way data are made available, used, and analyzed, but also as a catalyst for increasing leadership and prioritization of maternal and newborn health. The former provincial health director of Luapula Province noted, “Success of SMGL really grew out of improved information, audits and the ability to use data to hone in on problems and demonstrate that with planning and focused resources the problems could be addressed. We were able to see the results of our work and started asking other districts for similar information.”

In both Uganda and Zambia, maternal and perinatal death surveillance and review systems (MPDSR) were institutionalized and training programs and guidelines developed with the support of SMGL. Progress in MPDSR has been sustained in both countries in Phase 2 of SMGL. At the hospital level, for example, according to the HFA, 93% of hospitals in Uganda and 100% in Zambia conducted maternal death reviews in 2016. In Zambia, 75% of all deaths in SMGL districts were reported and reviewed in 2016. In focus group discussions, district leadership felt the process had become routine and could be sustained without external support. In both countries, non-healthsector leadership is now involved in death reviews, which continue to build political will and address maternal health appropriately within the broader community context, further adding to the likelihood of sustainability. In Zambia, district commissioners were appointed as heads of the district MDSR committee and required to submit their maternal death reports to the national government; this ensured a high level of political investment in reporting maternal deaths and in understanding the multiple causes of death. In Uganda, the birth weight and age-at-death boxes for an intervention and evaluation system (BABIES) matrix^28^—a simple data tool to better track and understand newborn deaths and stillbirths—facilitated understanding of the timing and causes of perinatal deaths through closely tracking fresh and macerated stillbirths and early neonatal deaths. It is now being used comprehensively in 6 of 13 SMGL districts and has been proposed in the Uganda MPDSR guidelines.

At the hospital level, 93% of hospitals in Uganda and 100% in Zambia conducted maternal death reviews in 2016.

SMGL strengthened Health Management Information System (HMIS) data in both countries through vigorous review of registers and health information aggregation reports by implementing partners with district health office staff. The data collection approach, implemented on a quarterly basis, included data collection from all possible data sources in a health facility across all existing departments—data that were later triangulated with the health information aggregation report. This worked as a validation process for HMIS and consequently improved site-level data collection and reporting. Ministry of Health district staff in Zambia commented that it became routine for SMGL team members to call and question them about their data quarterly. Consequently, district health workers took more interest and began reviewing data at the district headquarters before submitting, a process that continued after SMGL funding ended and is likely to be sustained. In Uganda, Pregnancy Outcome Monitoring Survey data were used to update the monthly maternity register summaries that were entered and reported into the national District Health Information System 2 database. This community data collection tool, which remains in place, was strengthened so that it could be used for pregnancy and MPDSR. SMGL supported procurement and supply of facility tools and data registers and facilitated orientation of health workers on the registers, if needed. One district health director in Zambia commented that the district would print registers if they were not available from the national level. This is not a universal position at the district level but, with continued external funding under the CoC program and heightened scrutiny at the national and provincial levels, these minor but critical tools are likely to remain in place for the foreseeable future.

In both Uganda and Zambia, the focus on routine data reviews beyond the facility resulted in routine, more meaningful, review meetings, where gaps were identified and addressed with decision makers in the room. These were simple but important problem-solving exercises. For example, a province in Zambia realized that a facility that had made only limited improvement in the number of antenatal care visits did not have a motorbike and reallocated one from an incoming shipment so the facility could conduct routine outreach to remote communities. This small data-based problem-solving process was repeated over time and helped address many health system issues that had previously seemed insurmountable. One district health director in Zambia noted that “as the SMGL metrics were absorbed into their performance standards, they [health care workers] became more accountable.” She felt this would be a key driver of sustainability.

## DISCUSSION

The rapid reduction in maternal mortality seen in Phase 1 of SMGL and the maintenance of these improvements, despite planned annual declines in external financial inputs during Phase 2, suggests that the health improvements demonstrated in SMGL-supported districts will be sustained. Despite uneven implementation following year 1 due to changes in U.S. Government implementing mechanisms in both countries and erratic funding flows, quarterly data reviews continued to yield positive results. In addition, because many lessons from SMGL have been incorporated into national policies and practice and have attracted support from other development partners and the private sector, these approaches will continue to be used and tested, at least in the immediate term. The challenge of inadequate human resources and low host-country financial investments, however, remain threats to further progress toward achieving long-term global and national development goals. Also, as frequently noted by local leadership, maternal mortality will continue to be affected by poverty, poor infrastructure, and weak education systems, which were not addressed by SMGL and will remain rate-limiting factors for improvement.

Despite uneven implementation following the first year, quarterly data reviews continued to yield positive results.

Gauging sustainability of the SMGL health systems approach against these domains can provide important insight into projected maternal and newborn outcomes as SMGL goes to scale in both countries. We suggest that addressing the following 4 questions can lay the groundwork for judging the impact of SMGL on sustainable improvements in the survival of mothers and newborns.

### Will There Be Sustained Impact on Demand for Safer Births?

We have described changes in community norms and behaviors that resulted in dramatic increases in facility delivery. Factors included involvement of community leaders as change champions, changing attitudes toward the role of men in birth planning, improved quality of facility delivery services, and increased accountability by political and health leadership in the outcomes of pregnancy. Learning from each tragic maternal or newborn death and improved trust and communication between communities and health workers can overcome the fear and fatalism that many perceive and help ensure continued progress in eliminating preventable deaths. From the baseline to endline census data, maternal mortality decreased by 41% in Zambia and 44% in Uganda in SMGL districts. These results aligned with trends in quarterly routine data collection throughout the implementation of SMGL.^33^

### Will Timely Access to Facility Births and EmONC Services Be Sustained?

Rural districts with deficient road and transportation infrastructure, high rates of poverty, and health facilities too few and far between were the settings for SMGL. Crucial to the initiative's success was an approach that focused on district system strengthening, which resulted in a variety of appropriate local solutions being developed. In Uganda, the use of transportation vouchers to utilize the available “Boda Boda” cyclists, the organization and coordination of ambulances (Box 3) to maximize efficiency, and upgrading facilities so that CEmONC functions were closer to people, all helped reduce the second delay. As a result, the met need for EmONC facilities increased by 65% in Uganda. In Zambia, the provision of bicycles, motorcycles, and ambulances, prioritizing pregnant women for use of these services; the construction and refurbishing of maternity waiting homes; and improved radio and mobile phone communication systems had similar effects during Phase 1, although by the end of Phase 2 the ability to provide services to meet demand had decreased by 11%. This result may suggest either that over the course of the SMGL initiative demand for EmONC services substantially increased through successful promotion of facility-based deliveries or that additional resources must be allocated to facilitate timely access to facilities. In either case, for both countries, district leadership and local resource allocation should continue to help guide and implement appropriate and efficient solutions. National leadership, governance, and better planning for adequate development and staffing of EmONC facilities are also crucial. General improvements in transportation and communication infrastructure and reductions in poverty may have the largest long-term impact on timely access to services. Making maternal and perinatal mortality a broader social priority and involving other sectors in planning, leadership, and accountability of related systems and services are also crucial to sustaining progress.

In Zambia, an 11% decrease in the ability to provide services over the course of the initiative suggests that additional resources must be allocated to facilitate timely access to facilities.

BOX 3 **Foundational Ambulance Systems in Established in Uganda**

Uganda did not have well-developed protocols for the organization of ambulance services. With SMGL support, district committees were organized and protocols adopted nationally. Several innovations included:

- Phone consultations for referral cases
- Development of ambulance teams
- Triplicate referral logbook
- On-call rooms for ambulance drivers
- Monthly and quarterly review of referrals and outcomes.

These helped to establish practices and policies, which have been adopted nationally. They will require modest support, which has been envisioned under existing activities for ongoing maintenance.

Abbreviation: SMGL, Saving Mothers, Giving Life.

### Will Quality Childbirth and Pregnancy Services Be Sustained?

As Dr. Jesca Nsunga Sabiiti, acting commissioner of community health in the Uganda MOH, stated, “counting deaths at district is the first step to better accountability.” Mentorship of midwives in both small and large health facilities played an important role in improving the skills and competence needed to respond quickly to obstetric complications and, perhaps more importantly, did not create gaps in service as off-site training often does. Although filling human resources gaps by hiring additional doctors and nurse-midwives was a necessary quick fix to ensure continuous availability of services at health centers, it cannot be sustained without improved national planning and budgeting for human resources and the willingness of the government to change retirement policies or create mechanisms for rehiring retired providers. Commodity security for essential drugs, especially uterotonics, at health centers and in communities; an improved supply of blood products; and more effective transfusion-prescribing practices will be crucial for reducing the leading cause of maternal mortality, obstetric hemorrhage, and will require greater national investment in national blood services and quality management of commodities to foster zero tolerance of stock-outs. SMGL demonstrated that other interventions, such as balloon tamponade, may also serve an important role in preventing maternal deaths, especially if CEmONC facilities are not easily accessible. SMGL also demonstrated the utility of partographs in improving the timeliness of referrals in obstetric emergencies and has helped to expand their continued use. We recognize, however, that SMGL was not fully successful in addressing all aspects of quality and that, in addition to considering sustainability of the approach, greater attention will be required to tackle adequate access to all aspects of EmONC, particularly cesarean delivery, and to addressing newborn mortality.

We believe that district-level commitment and leadership is crucial to ensuring that quality services are maintained. The introduction of MPDSR as standard practice, ensuring every death is counted and learned from, helps create and sustain a quality improvement culture. SMGL's contribution to MDSR and the BABIES matrix, and, specifically, the involvement of community leadership in these processes, is a best practice that can be replicated globally.

We believe that district-level commitment and leadership is crucial to ensuring that quality services are maintained.

The infrastructure improvements and equipment remaining in SMGL-supported health facilities will have a sustained impact in SMGL-supported districts. These investments have already paid dividends in lives saved and demand maintained, even beyond the target populations for the project. As a result, the described scale of the SMGL approach includes robust initial infrastructure investments. These improvements were based on careful facility assessments and addressed the specific needs of these districts. HFAs, while not a novel concept, should be an important part of other maternal, newborn, and child heath efforts and will require flexibility in external funding to address critical gaps that are identified—whether lack of an incinerator or staff housing or a weak supply chain for essential commodities. Ensuring that maintenance of facilities is continued and that EmONC facilities are located appropriately addresses issues of quality and access—2 of the delays targeted under SMGL. Institutionalization of services and maintenance of infrastructure also lend hope for the prospective sustainability of positive outcomes demonstrated by SMGL.

### Has SMGL Contributed to the Long-Term Strength and Resilience of the Health System?

The district systems strengthening approach made important contributions to the strength and resilience of the greater health systems in Uganda and Zambia, beyond just maternal and newborn health. For example, substantial increases in electricity, 24 hours-a-day/7 days-a-week service, transportation, and communications will have important ripple effects across the provision of all health care. However, for long-term sustainability, both countries will need to increase financial commitments for health as a proportion of their overall budgets.^8^ The benefits of SMGL included better coverage for HIV programs, especially for the prevention and elimination of mother-to-child transmission of HIV. Robust data collection and continuous learning and quality improvement will benefit other programs through better data quality and more complete health records. Pregnancy registration and identification of community maternal and newborn deaths will contribute to the transition to universal vital registration in resource-limited settings. Community health workers contributed to changing norms by providing health education to communities and involving fathers; by saving the lives of mothers and babies, they have increased their own value to their communities. Addressing transportation and communication issues for emergency services—including radio and ambulance systems as well as Boda Boda vouchers—can improve response to road traffic injury and other medical emergencies. The costing of services and linking them to outcomes has supported the expanded use of results-based financing and will improve the efficiency of financing. SMGL significantly catalyzed other donor-supported efforts, notably the World Bank program in both countries, Sida and DFID projects in Zambia, and Belgian-supported activities in Uganda. We hope these lessons will have a positive influence more broadly through the information shared in this supplement and elsewhere.

### Limitations

The most salient limitation of the methodology is that we are analyzing the likelihood of sustainability in the near– to mid-term and 5 or 10 years beyond the initiative. This is mitigated by the fact that SMGL used a declining fund model^2^ after the first year of the initiative and, unintentionally, there was a 1-year break in funding^2^ to both Uganda and Zambia during which core activities continued and results improved. The second limitation is that while we have confidence in the sustainability of the SMGL results, we do not believe the need for technical assistance, capacity building, or support has ended, and we have not analyzed in detail the type of continued support that will be required. Translating findings from HFAs into prioritized programming, for example, will require some level of technical assistance and capacity building for the near-term in both countries. As the SMGL approach is scaled up, it is imperative that such assistance—as is currently envisioned—provides this support. Finally, a limitation of assessing the sustainability of SMGL is that the initiative itself was not designed to be sustained, but rather to prove that reductions in maternal mortality were possible and introduce a sustainable approach to maternal and newborn mortality reduction for scale. The branding of the initiative as such may have unwittingly undermined country ownership of SMGL by linking it too directly to funding agencies—vs. the Ministries of Health—just as analyzing the sustainability of SMGL as an initiative may be seen to mask the true focus on the sustainability of a health systems strengthening approach to maternal mortality reduction. Similarly, aspects of the initiative were cumbersome and, we posit, could have been avoided by building on lessons from the SMGL experience. For example, the start-up of SMGL was rapid with a “build the plane as you are flying” mentality, which resulted in confusion at the community and district levels and, initially, planning and coordination challenges for partners.

## CONCLUSIONS

From the onset, in order to promote ownership and sustainability, SMGL was designed to reinforce host country government structures, policies, guidelines, and priorities. Strategic, long-term capital investments were made to enable districts to achieve national standards, including essential infrastructural renovations of health facilities and maternity waiting homes, provision of required equipment and supplies, training of medical personnel in critical lifesaving skills for CEmONC and BEmONC and mentorship, development of systems and procedures for verbal autopsies/maternal audits, and provision of ambulances. These investments represented a “big push” that was criticized^34^ as donor-driven and unsustainable but were deemed crucial to demonstrating the potential of the SMGL model. Following the capital investments, SMGL resources declined annually and implementation shifted to maintaining human and infrastructure investments. The early success of SMGL was a powerful contributor to building momentum and enthusiasm for the model and catalyzing scale, which continued to build through the life of the initiative. Will the level of scale achieved—covering over half of Zambia and three-quarters of Uganda—lead to improved and sustained health outcomes for mothers and newborns at national level? Will the results of SMGL be maintained and improved upon? Data from HFAs and multiple interviews in both countries suggest that increases in demand for quality services, access to care, and quality of care—through support from SMGL—have made a course change in focus districts and are likely to continue to reduce maternal and newborn mortality and morbidity. The SMGL theory of change has proved robust and the model successful, whether implemented directly by host-country ministries of health alone, in the case of select districts in Zambia, or with support from additional implementing partners. While we believe strongly in the potential of a systems approach to decrease maternal and newborn deaths, no approach can be effective without strong political will, at all levels, and a society's zero tolerance for preventable maternal and newborn deaths.

## Notes

**Peer Reviewed**

**Funding:** Saving Mothers, Giving Life implementation was primarily funded by the Office of the Global AIDS Coordinator, the U.S. Agency for International Development (USAID), Washington, D.C., the Centers for Disease Control and Prevention (CDC), Atlanta, Georgia (Cooperative agreements GPS003057 and GPS002918), Merck for Mothers, and Every Mother Counts. The funding agencies had no influence or control over the content of this article.
